# Effectiveness of an Individual Cognitive-Behavioral Intervention for Serious, Young Male Violent Offenders: Randomized Controlled Study With Twenty-Four-Month Follow-Up

**DOI:** 10.3389/fpsyt.2021.670957

**Published:** 2021-08-02

**Authors:** Martin Lardén, Jens Högström, Niklas Långström

**Affiliations:** ^1^Department of Clinical Neuroscience, Karolinska Institutet, Stockholm, Sweden; ^2^Swedish Prison and Probation Service, Norrköping, Sweden; ^3^Centre for Psychiatry Research, Department of Clinical Neuroscience, Karolinska Institutet, Stockholm, Sweden; ^4^Stockholm Health Care Services, Stockholm, Sweden; ^5^Department of Medical Epidemiology and Biostatistics, Karolinska Institutet, Stockholm, Sweden; ^6^National Board of Health and Welfare, Stockholm, Sweden

**Keywords:** violent crime, randomized controlled (clinical) trial, treatment outcome, reoffending, young offenders, cognitive behavioral therapy, residential treatment, aggression

## Abstract

**Background:** Psychological recidivism-reducing interventions with serious, young violent offenders in residential care have unsatisfactory effects. We tested if a complementary individual cognitive behavioral therapy (iCBT) intervention focusing problem-solving, cognitive self-control, and relapse prevention reduces criminal recidivism beyond usual institutional care encompassing interventions such as social skills training and prosocial modeling (treatment-as-usual; TAU).

**Method:** We consecutively approached 115 eligible serious, male violent crime offenders in five residential treatment homes run by the Swedish National Board of Institutional Care. Eighty-one (70%) 16 to 21-year-old youth at medium-high violent recidivism risk were included and randomized to an individualized 15 to 20-session CBT intervention plus TAU (*n* = 38) or to TAU-only (*n* = 43), 4–6 months before release to the community. Participants were assessed pre- and post-treatment, at 12 months (self-reported aggressive behavior, reconvictions) and 24 months (reconvictions) after release. Intent-to-treat analyses were applied.

**Results:** The violent reconviction rate was slightly higher for iCBT+TAU vs. TAU-only youth at 12 months (34 vs. 23%, *d* = 0.30, 95% CI: −0.24 to 0.84) and 24 months following release (50 vs. 40%, *d* = 0.23, 95% CI: −0.25 to 0.72), but neither of these differences were significant. Cox regression modeling also suggested non-significantly, negligibly to slightly more violent, and any criminal recidivism in iCBT+TAU vs. TAU-only youth during the entire follow-up. Further, we found no significant between-group differences in conduct problems, aggression, and antisocial cognitions, although both iCBT+TAU and TAU-only participants reported small to large within-group reductions across outcome measures at post-treatment. Finally, the 12-month follow-up suggested marginally more DSM-5 Conduct Disorder (CD) symptoms of “aggression to people and animals” in iCBT+TAU vs. TAU-only youth (*d* = 0.10, 95% CI: −0.40 to 0.60) although this difference was not significant.

**Conclusion:** We found no additive effect of individual CBT beyond group-based TAU in residential psychological treatment for serious, young male violent offenders. Limited sample size and substantial treatment dropout reduced the robustness of intent-to-treat effect estimates. We discuss the possible impact of treatment dose and integrity, participant retention, and TAU quality.

## Introduction

Interpersonal violence is a profound global social and public health problem. For instance, the World Health Organization (WHO) concludes that homicide is the third leading cause of death internationally for 15 to 44-year-old males (1). In a recent annual victim survey in Sweden, 3.5% of the population over 15 years reported physical assault victimization during 2018 (2) whereas 1.1% of US residents over 12 years described having been a victim of violent crime in 2019 (3). Considering the huge costs in human suffering and economic terms alike, even small reductions in violent crime is important [e.g., (4)]. In addition to broader universal and selective prevention efforts, effective treatment of identified, and convicted violent offenders is a vital component of a comprehensive violence prevention strategy. However, working with young in contrast to adult offenders requires attention to dissimilar judicial status, higher rates of antisocial behavior, and recidivism risk but also higher developmental malleability (5–7).

Providing effective recidivism-reducing interventions to young serious violent offenders, often in residential care, is a prioritized task for legal and social service authorities worldwide. However, placing antisocial youth in specialized residential treatment centers may have adverse effects, for example increased reoffending risk (8–10) and impaired adult physical and mental health (11). Many young offenders experience isolation and violations of their basic rights in institutions (12) and incarceration of young offenders may reinforce destructive behaviors (13). For instance, through attention and encouragement from peers when exhibiting oppositional or aggressive behaviors toward staff. Such negative influences or *contagion effects* suggest a need for individualized interventions to complement the more common group-based interventions in juvenile forensic institutions (12, 14).

Although treatment effects tend to be small, systematic reviews have suggested promising treatments to reduce criminal recidivism [e.g., (5, 15)]. Regarding young offenders, Armelius and Andreassen (16) systematically reviewed 12 randomized controlled trials (RCTs) and non-randomized controlled trials of interventions based on cognitive behavioral therapy (CBT) targeting 12 to 22-year-old incarcerated young offenders. Cognitive behavioral therapy-based interventions were associated with a small recidivism risk reduction (10% on average) in any new crime at 12-month follow-up compared to controls. In contrast, no significant treatment effects were found at 6 and 24 months, nor did data suggest differences across different CBT interventions. Morales et al. (4) conducted a systematic review of 31 randomized or quasi-experimental studies of 12 to 21-year-old offenders incarcerated for serious or repeated violent or non-violent offending. Their findings suggested marginal reductions of violent and general recidivism (odds ratio = 1.27, *p* = 0.005) for cognitive behavioral and multimodal interventions. Compared to control groups, Koehler et al. (17) found CBT interventions to be more effective (mean reduction 13%) in reducing reoffending than non-CBT interventions (mean reduction 6%) in a systematic review of treatment programs in Europe for offenders <25 years of age. In a meta-analysis of 27 primary controlled studies, De Swart et al. (10) compared the effectiveness of broadly defined *evidence-based institutional treatment* with other forms of institutional and non-institutional care with at least post-treatment measures as outcome. Their results indicated that institutional care could be as effective as non-institutional care, and that evidence-based interventions on average proved more effective than institutional care-as-usual (*d* = 0.34). Specifically, CBT-interventions had a moderate effect (*d* = 0.50) based on a summary measure containing delinquency, behavior problems, skills, and a miscellaneous problem category.

Importantly, a meta-analysis of six studies with 13 effect sizes by Hoogsteder et al. (18) suggested that interventions with *individualized CBT components* could be more effective in reducing severe aggressive behavior in adolescents compared to regular care or treatment-as-usual (TAU) with no CBT components (between group *d* = 1.14). The authors conclude that the addition of individually tailored interventions based on the *risk, needs, and responsivity* (RNR) principles (19) to group interventions might improve outcome for aggressive adolescents.

The rationale for this study was the weak effects found previously for interventions administered in routine practice targeting incarcerated serious, young male violent offenders with medium to high recidivism risk. Hence, we attempted to improve the existing evidence base by conducting a five-site RCT in Sweden to evaluate the effectiveness of an individual, manualized CBT intervention (iCBT) added to standard group-based treatment (TAU) in reducing reoffending, compared to TAU alone. Specifically, our hypothesis was that iCBT+TAU would reduce self-reported conduct problems, aggression, and antisocial cognitions as well as criminal recidivism more than TAU-only.

## Materials and Methods

### Setting

We conducted a randomized, controlled trial in Sweden across five (out of the six existing) residential facilities for serious young violent offenders sentenced by general criminal courts to secure care according to the Closed Institutional Youth Care Act. The secure facilities, administered by the National Board of Institutional Care, are located across Sweden and participant inclusion occurred between December 2003 and August 2006. Sweden has no formal separation of juvenile and adult justice systems and Closed Institutional Youth Care was introduced on January 1, 1999, as a new sentence for adolescents between 15 (age of criminal responsibility) and 17 years guilty of serious criminal offenses. Crime categories usually include (aggravated) robbery or assault, homicide or rape. Closed Institutional Youth Care was introduced as a custodial replacement to imprisonment with adults in the general prison system. Sentence lengths vary from 14 days to 48 months, the full term is served in an institution and there is no parole. During 2000–2006, when the youth in this study were convicted, 115–120 individuals yearly (97% male) were convicted to an average of 9 months in secure care according to the Closed Institutional Youth Care Act (20). For a detailed account, see also Pettersson (21).

### Participants

We included young male offenders with 4–6 months remaining of an ongoing residential youth care sentence of ≥6 months for a non-sexual, violent crime. We defined violent offenses as homicide, assault, assault of an officer, robbery, and aggravated arson while sexual offenses were not included. Attempted or aggravated versions of these offenses were included whenever applicable. Female offenders were not included due to very small numbers overall and placement in non-participating residential facilities. Youth were ineligible to participate if they did not speak Swedish sufficiently well or had current severe, destabilizing psychiatric disorders involving psychotic or suicidal features.

Twenty-one youth were lost from possible participation prior to being asked. Eleven of these were either moved to a non-participating institution or available psychotherapist(s) had no room at the time to take them on for iCBT following possible inclusion. Another ten individuals were not asked due to miscommunication between researchers and staff at the five participating institutions. Finally, four youth were not approached due to intellectual impairment (total IQ <70) as ascertained from psychological testing or a psychiatrist or psychologist's clinical judgment.

**Figure 1:**
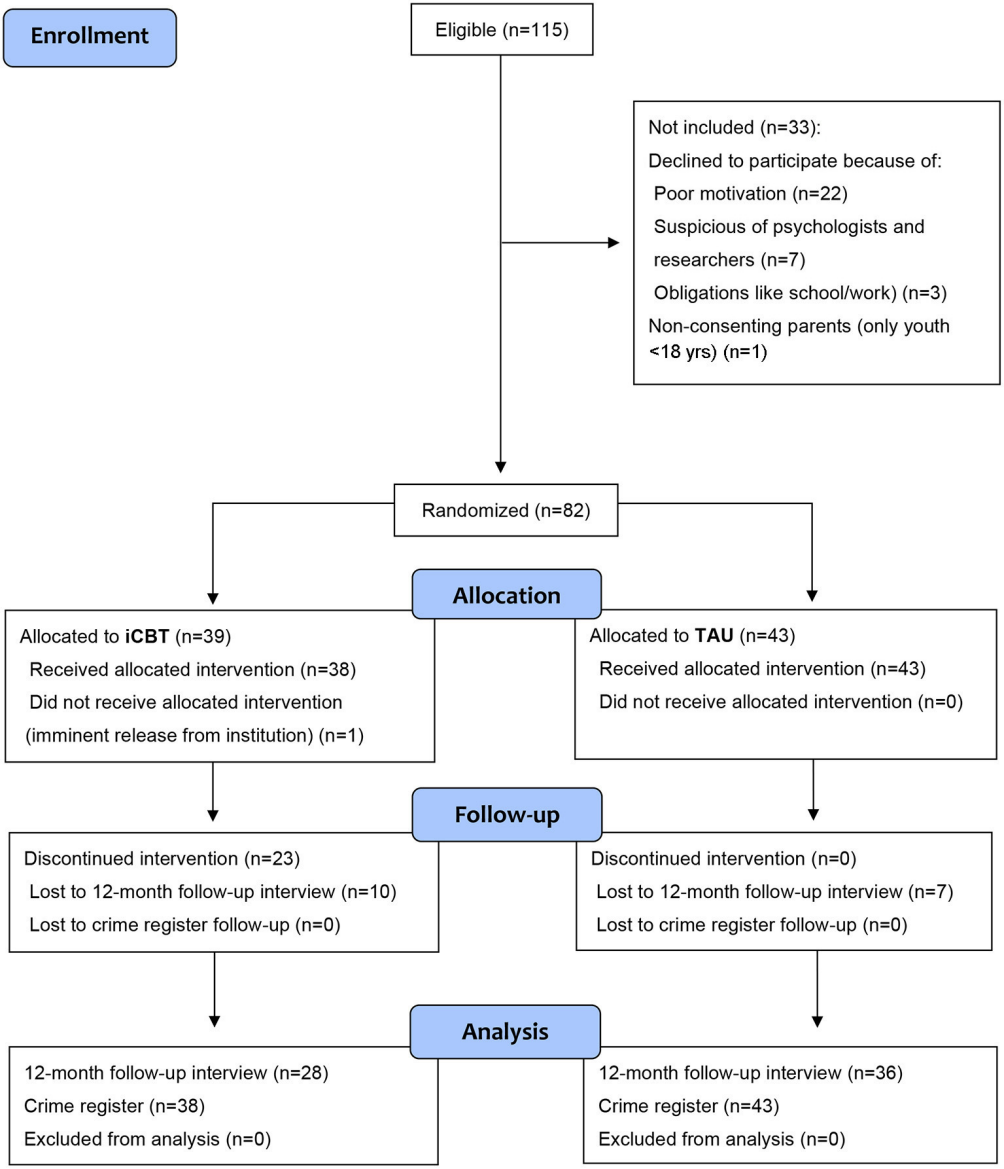
Flowchart of a randomized controlled trial (RCT) comparing an individualized cognitive-behavioral intervention (iCBT) plus group-based treatment-as-usual (TAU) with TAU-only among serious, young male violent offenders.

A total of 115 eligible male youth were asked for possible inclusion, 82 of which (71.3%) agreed to participate following oral and written informed consent (see Figure 1). Main self-reported reasons for non-participation included poor motivation and being suspicious of psychologists and researchers. Participants (*M* = 17.7, *SD* = 0.9) were moderately younger than non-participants (*M* = 18.3, *SD* = 1.0, *p* < 0.01). The proportion of youth that consented to inclusion ranged from 67% (18/27) to 74% (17/23) across the five residential treatment homes. The average sentence length for participating youth was 10.2 months (*SD* = 6.3, range: 5.0–48.0). Since one male was mistakenly asked to participate just before release, 81 subjects were randomized either to TAU+iCBT (*n* = 38) or TAU-only (*n* = 43); the difference in numbers was due to chance.

#### Sociodemographic Characteristics

Participants were all males aged 16–21 years at inclusion. Twenty-five percent (*n* = 20) were non-immigrants with majority ethnicity, defined as being born in Sweden with both biological parents born in Sweden. Forty-six percent (*n* = 37, including two trans-nationally adopted boys) were first-generation immigrants, born abroad and with both parents born abroad. Thirty percent (*n* = 24) were second-generation immigrants, born in Sweden with one or both parents born abroad. For further details, see Table 1.

**Table 1:** Baseline sociodemographic and pre-treatment data for participants in an RCT of an individualized CBT intervention (iCBT) plus treatment-as-usual (TAU) vs. TAU-only among convicted serious, young male violent offenders.

**Baseline characteristic**	**iCBT participants (*n* = 38)**	**TAU participants (*n* = 43)**	**Effect size for difference Cohen's *d***
**Sociodemographic variables**			
Age at inclusion, years, *M, SD*	18.0 (0.9)	17.7 (0.7)	*0.29^*ns*^*
Urban area of residence, % (*n*)	45% (17)	67% (29)	–0.47^*^
**Migrant status**			
Born in Sweden w both parents born in Sweden, % (*n*)	32% (12)	19% (8)	
Born in Sweden w one parent born abroad, % (*n*)	29% (11)	30% (13)	–*0.31^*ns*^*
Born abroad, % (*n*)	40% (15)	51% (22)	
**Index offense**			
(Attempted) homicide, % (*n*)	11% (4)	5% (2)	
(Aggravated) assault, % (*n*)	29% (11)	26% (11)	–*0.26^*ns*^*
(Aggravated) robbery, % (*n*)	58% (22)	65% (28)	
Aggravated arson, % (*n*)	3% (1)	5% (2)	
Length of index sentence, months, *M, SD*	10.53 (7.27)	10.00 (5.36)	0.08*^*ns*^*
**Residential treatment home**			
A, % (*n*)	26% (10)	23% (10)	
B, % (*n*)	24% (9)	23% (10)	
C, % (*n*)	21% (8)	23% (10)	−0.05*^*ns*^*
D, % (*n*)	18% (7)	21% (9)	
E, % (*n*)	11% (4)	9% (4)	
**Psychological functioning, aggression, and social cognition**			
Youth Self-Report, affective^a^ (0–24), *M, SD*	4.89, 4.05	4.77, 3.21	0.04*^*ns*^*
Youth Self-Report, anxiety^a^ (0–12), *M, SD*	2.45, 1.80	2.35, 2.15	0.05*^*ns*^*
Youth Self-Report, somatic^a^ (0–14), *M, SD*	2.39, 2.49	1.91, 1.94	*0.22^*ns*^*
Youth Self-Report, ADHD^a^ (0–10), *M, SD*	3.29, 2.51	3.58, 1.99	−0.13*^*ns*^*
Youth Self-Report, oppositional defiant^a^ (0–10), *M, SD*	3.71, 2.75	3.91, 2.24	−0.08*^*ns*^*
Youth Self-Report, conduct problems^a^ (0–28), *M, SD*	7.53, 4.90	6.79, 3.64	0.17*^*ns*^*
Aggression, total^b^ (0–42), *M, SD*	11.18, 7.37	10.07, 6.24	0.16*^*ns*^*
Aggression, proactive^b^ (0–20), *M, SD*	5.18, 4.19	4.65, 3.27	0.14*^*ns*^*
Aggression, reactive^b^ (0–12), *M, SD*	3.55, 1.91	3.28, 1.94	0.14*^*ns*^*
Antisocial cognitions^c^ (40–240), *M, SD*	121.50, 43.50	113.79, 28.27	*0.21^*ns*^*
Socio-moral reflection ability, total score^d^ (11–33), *M, SD*	16.97, 3.73	17.81, 3.78	−0.08*^*ns*^*
**Substance abuse**			
SAVRY item 19^e^ % (*n*)			
Low	26% (10)	21% (9)	
Medium	18% (7)	40% (17)	0.12*^*ns*^*
High	55% (21)	40% (17)	
**Psychopathic personality traits and recidivism risk**			
PCL:SV, total score (0–24), *M, SD*	12.47, 4.66	12.95, 5.34	−0.10*^*ns*^*
PCL:SV, interpersonal/affective factor (0–12), *M, SD*	4.79, 3.02	5.37, 3.30	−0.18*^*ns*^*
PCL:SV, unstable lifestyle/antisocial factor (0–12), *M, SD*	7.89, 2.19	7.79, 2.62	0.04*^*ns*^*
SAVRY, total risk score^f^ (0–48), *M, SD*	23.42, 6.26	22.33, 7.62	0.16*^*ns*^*
SAVRY, overall risk^g^, % (*n*)			
Low	10% (4)	12% (5)	
Medium	58% (22)	44% (19)	−0.18*^*ns*^*
High	32% (12)	44% (19)	
SAVRY, total protective score^h^ (0–6), *M, SD*	3.34, 1.48	2.84, 1.82	*0.30^*ns*^*

*Following Cohen (22): Italicized figures denote 0.20 < d < 0.50, equal to a “small” effect size*.

a *DSM diagnosis-oriented subscale, past 6 months*.

b *No specified period*.

c *Self-reported antisocial cognitive distortions during past 6 months according to How I think, total score. Higher scores indicate more distortions*.

d *Higher score indicates more mature socio-moral judgments*.

e *SAVRY item 19, Substance use difficulties, refers to alcohol or drug use that is sufficiently severe to cause problems in physical health or in one or more major areas of functioning*.

f *Summary risk score across all 24 SAVRY risk items rated 0 = low, 1 = medium, or 2 = high*.

g *Distribution of overall structured professional recidivism risk judgments over low, medium, and high risk*.

h *Summary score across all six protective factors rated 0 = absent or 1 = present*.

ns *p > 0.05*,

* *p < 0.05*.

### Procedure

The Regional Research Ethics Committee in Stockholm approved of the study (dnr: 03–315). Eligible young violent offenders at the five participating residential treatment facilities were consecutively asked to participate by a psychotherapist or another staff member, usually the head of the ward. Subjects provided oral and written informed consent to participate. For participants younger than 18 years, oral and written permission were also obtained from his legal guardian. At baseline, the youth completed six self-report questionnaires (described below) provided by the staff at the residential facilities. Participants were informed that all information they provided during baseline assessments and throughout the study was for research only and would not be accessible for ward and clinical staff. The youth were given as much time they needed for the completion of the questionnaires and instructed to ask a nearby staff member for help in case of difficulty to read or understand items. The staff made sure that the youth completed the questionnaires on his own, with no other youth disturbing him. Each participant personally put his filled-out questionnaires in an envelope and sealed it followed by the envelope being collected by the research assistant. Within a week from the completion of the questionnaires, the youth was interviewed and assessed face-to-face with the SAVRY and the PCL:SV by a trained research assistant (B.Sc. in psychology and criminology). The research assistant was not involved in youth care and all participants were again carefully informed that no information provided during assessments would be revealed to ward staff. We also explicitly informed about the only exception to this: a duty to report to the social services (according to the Swedish Social Services Act) if the youth would reveal information about any specific, named child currently at imminent risk of suffering harm, including child abuse or neglect. No such reporting was deemed necessary during the trial.

Participants received 100 SEK (approximately 10.50 USD) for completion of self-report questionnaires and participation in the baseline interview. One to three weeks before leaving the institution, the youth completed post-treatment questionnaires, again administered by the staff. Again, we reminded participants that all information was for research only and would not be used against them. Subjects received another 100 SEK upon completion of the post-treatment assessment.

### Randomization

Participants were randomized to either iCBT plus the standard intervention (TAU) (experiment group) or TAU-only (control group) across all five sites to reduce the risk of bias due to intervention differences between units. Randomization was obtained with a pre-existing, computer-generated series in an unweighted fashion with either “iCBT” or “TAU” printed on paper at an overall 1:1 ratio. Every single printout was individually placed in opaque envelopes held centrally by the research group and later drawn by the last author when contacted by the research assistant or a site coordinator reporting that a specific eligible youth had completed pre-trial assessments.

### Interventions

#### Individual Cognitive Behavior Therapy

Individual Cognitive Behavior Therapy (iCBT) (23) is a manualized treatment program for serious violent offenders, developed by the first author, and based on extant research on violent offending and evidence-based treatment of serious young offenders at the beginning of the 2000s (24–26). Andrews' and Bonta's (27) influential textbook *Psychology of criminal conduct* provided important inspiration for the program, particularly regarding adherence to the RNR principles of effective rehabilitation. These principles suggest that effective treatments against antisocial behavior should focus on offenders with higher recidivism risk, target criminogenic needs that drives criminal behavior, and address individual learning styles as well as resources and barriers within and around the individual offender responsivity (19).

Social learning and cognitive behavioral theories and methods as well as treatment philosophy were described in detail in Lardén (28), that was mandatory reading for treatment providers together with the formal iCBT manual (23). The main purpose of iCBT is to enhance adolescents' prosocial skills by practicing newly learned problem-solving and cognitive self-control strategies to manage everyday situations at the institutions. In a relapse prevention strategy, these new skills are hereafter adapted and planned for use in post-release real-life situations. Relapse prevention also included interpersonal skills training and identification of social network persons who could function as prosocial support after release. The iCBT intervention has four main phases aimed to strengthen prosocial skills and reduce recidivism risk: motivation and goal setting, social problem-solving training, cognitive self-control training, and relapse prevention. A complete iCBT intervention comprises 15–20 individual 45-min one-to-one sessions, administered approximately once per week.

An individual case formulation based on identified criminogenic needs was conducted at the start of the intervention. The case formulation contained a description of the adolescent's criminal history focusing on both the index offense and prior antisocial development. First, five criminogenic need domains related to recidivism risk (19) were mapped: persistence and pervasiveness of antisocial behavior, antisocial attitudes and values, substance misuse, temperament and personality factors that influence antisocial behavior, as well as psychiatric morbidity related to antisocial behavior. Second, antisocial peers and associates involved in or supportive of the adolescent's antisocial behavior around the index crime were identified, as well as persons who could function as prosocial role models. Prior experiences from school and vocational training were also described. Finally, the adolescent's strengths and resources that could protect against recidivism or enhance treatment progress were listed. The case formulation was a basis for the idiographic iCBT content delivered according to 15 manualized sessions (see Appendix 1 in Supplementary Material). Depending on the adolescent's specific needs and responsivity, some sessions were repeated up to three times.

For the iCBT group, the intervention was added to ordinary treatment curricula at participating institutions, while controls received solely the ordinary curricula (TAU). Seven therapists (4 men and 3 women) were recruited from the treatment staff at participating institutions (mean age = 49.3, range 20–54 years). Three were board-certified clinical psychologists, two academic social workers, and two were staff with a general education and specific training in individual psychotherapy. All, except one psychologist, had more than 10 years' experience of clinical work with serious young offenders. All therapists/iCBT providers attended an initial 2-day iCBT training seminar. Treatment integrity was upheld through repeated, individual supervision by e-mail up to once per month and through 1- or 2-day meetings twice a year.

#### Treatment-As-Usual

Treatment-as-usual consisted of ordinary residential treatment interventions at the five participating institutions; the specific contents varied across sites. Most of the time in residential homes was spent on structured activities of daily living, formal education, and leisure activities. The most common active intervention was interpersonal skills training sessions based on Aggression Replacement Training (ART) (29) with up to weekly group-based sessions. Other common interventions included ART-based anger management training, usually with group sessions once weekly for 10 weeks and supportive family therapy/network meetings with the individual young offender and his family members. To ascertain similar intensities of the TAU condition for both iCBT+TAU and TAU-only youth, we reminded the sites to maintain the ordinary treatment plan whenever a new participant was included in the study. No other individual psychological treatments took place during the study period. Apart from occasional medication with SSRIs, pharmacological treatment with antipsychotics, mood stabilizers, stimulants, and medications against substance misuse was uncommon in residential treatment at the time of the study.

### Measures

#### Self-Report Questionnaires

##### Youth Self-Report

The *Youth Self-Report* (YSR) (30) is a 111-item self-report questionnaire for 11 to 18-year-olds that taps emotional and behavioral problems dimensionally. Youth respond about the past 6 months on a three-point scale: *0* = *not true, 1* = *somewhat or sometimes true, and 2* = *very true or often true*. Studies suggest that the DSM-oriented subscales of the YSR have acceptable validity (31, 32). The YSR has been translated and validated in Sweden and Swedish validation data suggest acceptable to good internal consistency for the three tested affective-, anxiety-, and attention problem scales for boys aged 13–18 years [Cronbach's α ≥ 0.70, (33)]. We used YSR's six DSM-oriented subscales: *affective-, anxiety-, somatic-, ADHD-, oppositional defiant, and conduct problems* for baseline assessments and specifically oppositional defiant and conduct problems for pre-post treatment comparisons.

##### Reactive and Proactive Aggression Scale

We used a self-report version of the *Reactive and proactive aggression* scale (34) tapping two subtypes of aggressive behavior. The instrument contains 21 items; 10 measures proactive aggression and 6 reactive aggression while 5 are neutral items not loading on either scale. An example item is “*Threatens others.”* Subjects respond on a three-point Likert-type scale (*0* = *never, 1* = *sometimes or 2* = *often*) and items are added in a linear and unweighted fashion to subscale summary scores. The zero-order correlation between the 10-item proactive and 6-item reactive aggression scales was high in the original version (*r* = 0.70). Internal consistency was also high (Cronbach's α = 0.94 and 0.92, respectively). In this study, internal consistency was high for the total and proactive scales (α = 0.81 and 0.85, respectively), but weaker for the reactive scale (α = 0.57). We used the total score, as well as proactive and reactive aggression subscales for baseline and post-treatment assessments.

##### How I Think

*How I Think* (HIT) (35) is a 54-item self-report questionnaire addressing self-serving cognitive distortions. It contains 39 items tapping attitudes or beliefs related to antisocial behavior, 8 items to control anomalous responses, and another 7 items are positive fillers. Subjects respond on a six-point Likert scale (from *1, I agree strongly* to *6, I disagree strongly*) where high scores indicate more cognitive distortions. Internal consistency expressed as Cronbach's α was 0.94 in this study, compared to 0.96 in a previous Swedish report with adolescents as well as for the original English version (35, 36).

##### Sociomoral Reflections Measure—Short Form

*Sociomoral Reflections Measure*—*Short Form* (SRM-SF) (37) is a self-report instrument addressing moral judgement development according to the *neo-Kohlbergian typology*. The SRM-SF contains brief contextual statements and moral evaluation questions. Subjects evaluate and justify how important it is to act in a certain way according to 11 open-ended questions. Response patterns are evaluated and coded by an expert rater according to the SRM-SF manual. Studies suggest acceptable reliability and validity of such coding in youth (37–39), including good internal consistency (Cronbach's α = 0.93) and inter-rater reliability for the total score (ICC = 0.83) (37), expressed with the single rater intraclass correlation coefficient (ICC) (40). The Swedish version exhibited similar good inter-rater reliability (single rater ICC = 0.82) in a previous study of antisocial and matched general population adolescents (36). For the current sample, we found good internal consistency (α = 0.79) and inter-rater reliability for total scores (single rater ICC = 0.83).

#### Baseline: Expert Ratings

##### The Structured Assessment of Violence Risk in Youth

*The Structured Assessment of Violence Risk in Youth (*SAVRY) (41) is a risk assessment protocol based on the structured professional judgment model and includes ten historical risk factors, six social/contextual, eight individual risk factors, and six protective factors. Risk factors were coded on a three-level ordinal scale as *low (0), medium (1)*, or *high (2)* while protective factors were coded dichotomously as *present (1) or not (0)*. SAVRY was translated into Swedish by the last author following the North American original as closely as possible and yet being sensitive to Swedish social and legal conditions. We made a minor adjustment regarding the final professional judgment of future violence risk by excluding sexual crime from recidivism that the rater should aim at predicting, as risk factors for sexual reoffending in adolescents are partly different from those covered by SAVRY (42–45). We summed the ratings of the 24 historical, social/contextual, and individual risk factors, resulting in total SAVRY risk scores ranging from 0 to 48. Interrater reliability for the SAVRY summary risk score, obtained from 25 joint sit-in ratings by two trained, independent raters, was an excellent single rater ICC = 0.92.

##### Psychopathy Checklist: Screening Version

The Psychopathy Checklist: Screening Version (PCL:SV) (46) was developed from the original Psychopathy Checklist-Revised (47) to screen for possible psychopathy. The PCL:SV is validated for use with individuals from age 16. The PCL:SV consists of 12 items based on the 20-item PCL-R. Each item of the PCL:SV is scored on a three-point ordinal scale; *not present (0), partly/maybe present (1)*, or *definitely present (2)*. Inter-rater reliability, again measured across 25 individuals, was high (single rater ICC = 0.81) for PCL:SV total scores and interpersonal/affective and unstable lifestyle/antisocial factors had interrater reliability scores of 0.81 and 0.68, respectively. Whether violent offenders with many psychopathic personality traits are truly treatable has been an important question in correctional and forensic practice [e.g., (48–50)]. Although components of expert-rated psychopathy (according to PCL:R/-SV) beyond antisocial lifestyle tend to be unrelated to violent recidivism, we tested if PCL:SV psychopathy differed between youth randomized to iCBT+TAU and TAU-only. However, no baseline differences were found, and psychopathy was not used as a moderator variable.

### Outcome

#### Aggressive Behavior

Aggressive behavior at 12-month follow-up was measured as a conduct disorder (CD) symptom summary score derived from structured questions in follow-up telephone interviews with each participant's social service case manager, the youth himself, or both. The interviewing research assistant was masked to the youth's prior residential treatment allocation (iCBT+TAU or TAU-only) and participants and social service case managers were explicitly instructed at the beginning of the interview to not tell the interviewer details about prior residential treatment. We obtained interpretable data from 58 youth interviews and 49 interviews with social service staff. When both sources were available (*n* = 43), we used the highest reported value. The outcome score was based on an unweighted summary of the seven^1^ aggressive CD symptoms in DSM-5 (51). Interview responses were provided on a five-point scale (*never, 1–2 times, 3–5 times, 6–10 times*, and *11*+ *times*) regarding the past 12 months (i.e., from the end of treatment to the day of the interview). We recoded answers into a three-point scale: (*0* = *never, 1* = *1–2 times, 2* = *3*+ *times*) resulting in a possible score range of 0–16. Aggressive CD symptom data were provided by 64 of the 81 participants (79%). Ten iCBT participants and seven TAU-only controls were unavailable for this outcome.

#### Register-Based Criminal Reconvictions

We also addressed registered criminal re-offending during follow-up leading to a conviction registered in the National Crime Register held by the Swedish National Council for Crime Prevention (2). Data for this outcome were obtained for all participants until December 31, 2008.

Violent recidivism included homicide, assault, violence against an officer, robbery, and aggravated arson. Aggravated and attempted versions of these offenses were also included whenever applicable. Crime Register data reflect that the Swedish judicial system does not allow for plea bargaining so violent crime charges are never pleaded down, precluding loss of cases due to plea bargaining. Further, the Swedish legal system convicts individuals as guilty regardless of the presence of any psychiatric disorder, although sentencing might be informed by such disorders.

*Any criminal recidivism* included reconvictions for all violent offenses listed above but also gross violation of a woman's/person's integrity, illegal coercion, illegal threats, and intimidation, rape and other sexual crimes, and all other offenses according to the Swedish Penal Code and Narcotics Act. The nationwide National Crime Register provided criminal reconviction data for violent and general crimes, respectively, at 12- and 24-month follow-ups. We addressed *frequency of reoffending* as the count of new registered crimes across separate court sentences committed at 12- and 24-month follow-up, respectively.

### Statistical Analysis

We computed Cohen's *d:s* with 95% confidence interval as effect size measure with the freely available Practical Meta-Analysis Effect Size Calculator provided by the Campbell Collaboration [(52), based on (53)]. Following Cohen (22), *d*:s were interpreted as marginal (<0.20), small (0.20–0.49), moderate (0.50–0.79), or large (0.80+) effects. Pre- to post-test comparisons were done variable-wise as paired *t*-tests that were translated into Cohen's *d*:s using the freely available effect size calculator provided by the Memorial University of Newfoundland, Canada (54).

For pre- to post-treatment changes across six outcomes (Table 2), we used a mixed-effects ANOVA with group (iCBT vs. TAU-only) entered as a fixed effect and time (pre- vs. post-measurement) as a random effect in a repeated measures design. Missing data, usually less than five data points but occasionally up to ten within one subject, were handled by single mean imputation. For registered criminal recidivism during the entire follow-up period, we used Cox regression modeling with five empirically plausible covariates (age, urban residence, migrant status, antisocial cognitions, and SAVRY protective factors) with baseline differences of *d* ≥ 0.20). All statistical analyses were performed with the IBM Statistical Package for the Social Sciences (SPSS) version 24.

**Table 2:** Pre- to post-treatment comparisons (within-group) of self-reported conduct problems, aggression, and antisocial cognitions in an RCT of an individualized CBT intervention (iCBT) plus treatment-as-usual (TAU) vs. TAU-only among serious, young male violent offenders.

**Variable**	**iCBT participants**		**Effect size for difference Cohen's *d***	**TAU participants**		**Effect size for difference Cohen's *d***
	**Pre-treatment**	**Post-treatment**		**Pre-treatment**	**Post-treatment**	
Youth Self-Report, oppositional defiant^a^ (0–10), *M, SD* (*n*)	3.71, 2.75 (38)	1.27, 1.01 (33)	**0.94** ^***^	3.91, 2.24 (43)	1.33, 0.92 (40)	**1.12** ^***^
Youth Self-Report, conduct problems^a^ (0–28), *M, SD* (*n*)	7.53, 4.90 (38)	1.36, 1.85 (33)	**1.40** ^***^	6.79, 3.64 (43)	1.10, 1.34 (40)	**1.64** ^***^
Aggression, total^b^ (0–42), *M, SD* (*n*)	11.18, 7.37 (38)	8.34, 6.27 (32)	**0.62** ^***^	10.07, 6.24 (43)	7.75, 6.77 (40)	0.39^*^
Aggression, proactive^b^ (0–20), *M, SD* (*n*)	5.18, 4.19 (38)	3.81, 3.46 (32)	**0.65** ^***^	4.65, 3.27 (43)	3.78, 3.39 (40)	*0.23* ^ns^
Aggression, reactive^b^ (0–12), *M, SD* (*n*)	3.55, 1.91 (38)	3.37, 1.79 (32)	0.02^ns^	3.28, 1.94 (43)	2.67, 2.09 (40)	*0.26* ^ns^
Antisocial cognitions^c^ (40–240), *M, SD* (*n*)	121.50, 43.50 (38)	108.44, 41.98 (32)	*0.34* ^§^	113.79, 28.27 (43)	105.37, 40.36 (40)	*0.27* ^§^

*(n) denotes number of subjects with data in each treatment condition. Following Cohen (22): Bolded figures denote d > 0.50, equal to a “moderate” or larger effect size, italicized figures denote 0.20 < d < 0.50, equal to a “small” effect size*.

a *DSM diagnosis-oriented subscale, past 6 months*.

b *No specified measurement period; asks about how well the 21 statements agree with how you are*.

c *Self-reported antisocial cognitive distortions, past 6 months according to How I think, higher scores indicate more distortions*.

ns *Paired t-test, p > 0.10*,

§ *Paired t-test, p < 0.10*,

* *Paired t-test, p < 0.05*,

*** *Paired t-test, p < 0.001*.

### Power Analysis

Based on our reading of the literature when planning the study, we assumed a, in hindsight overly optimistic, difference in violent recidivism rates of 50 vs. 25% for iCBT+TAU over TAU-only. Before starting the study, we decided on a less conservative α of 0.10 in an attempt to balance risks for false positive findings (type I errors) and false negatives (type II errors). Reaching statistical significance at α = 0.10 (two-sided test) with a power of 0.80 would require 88 (44 treated and 44 control) participants.

## Results

### Baseline Comparisons of iCBT and TAU-Only Participants

Table 1 displays baseline data for participants and reveals a few small-sized (*d* = 0.21–0.47), significant (urban residence) and non-significant baseline differences (age, migrant status, index violent offense type, somatic anxiety, antisocial cognitions, and SAVRY protective factors) between youths randomized to iCBT+TAU or TAU-only. Except for somatic anxiety and index violent offense type, both empirically unlikely to be related to violent recidivism risk, five of these seven covariates were controlled for in the Cox regression model described below.

### Pre- to Post-treatment Comparisons

Pre- to post-treatment reductions in self-reported conduct problems, aggression, and antisocial cognitions are presented in Table 2. Both iCBT+TAU and TAU-only youth reported significant small-to-large effect-size reductions on all six measures, except for reactive aggression for iCBT+TAU participants. No between-group or interaction effects were found using mixed-design ANOVAs except for proactive aggression where iCBT+TAU participants reported a tendency toward more self-reported improvement [*F*_(1, 70)_ = 2.99, *p* < 0.10].

### Follow-Up: Aggression at 12 Months

The mean DSM-5 CD aggressive symptom score was 4.27 (*SD* = 3.55, range 0–11). We found no significant difference in CD symptom scores between iCBT+TAU and TAU-only participants (*t* = 0.39, *df* = 62, *p* = 0.70, *d* = 0.09) as reported by the youth themselves or their social service case managers (see Table 3).

**Table 3:** Aggressive symptoms and register-based criminal reconvictions at follow-up in an RCT of an individualized CBT intervention (iCBT) plus treatment-as-usual (TAU) vs. TAU-only among convicted serious, young male violent offenders.

**Outcome**	**12-month follow-up**			**24-month follow-up**		
	**iCBT participants(*N* = 38)**	**TAU participants(*N* = 43)**	**Effect size difference Cohen's *d* (95% CI)**	**iCBT participants(*N* = 38)**	**TAU participants(*N* = 43)**	**Effect size difference Cohen's *d* (95% CI)**
Aggressive DSM-5 CD symptom score^a^ (0–16), *M, SD*	4.46, 3.67 (28)^b^	4.11, 3.50 (36)^b^	0.10 (−0.40 to 0.60)	NA	NA	NA
**Criminal reconvictions**						
Violent crime^c^, % (*n*)	34% (13)	23% (10)	*0.30 (*–*0.24 to 0.84)*	50% (19)	40% (17)	*0.23 (*–*0.25 to 0.72)*
Any crime, % (*n*)	71% (27)	65% (28)	0.15 (−0.37 to 0.67)	71% (27)	74% (32)	−0.09 (−0.63 to 0.45)
No. of offense count^d^, *M, SD*	5.61, 8.95	3.49, 4.31	*0.31 (*–*0.13 to 0.74)*	7.82, 9.05	5.14, 6.35	*0.35 (*–*0.78 to 0.10)*

*Following Cohen (22): Italicized figures denote 0.20 < d < 0.50, a “small” effect size*.

a *Summary score of eight possible DSM-5 Conduct Disorder symptoms scored 0 = never, 1 = 1–2 times or 2 = 3+ times that the participant had acted accordingly during the past 12 months. Based on masked researcher interviews with each participant's social service case manager, the youth himself or both*.

b *Figures within parentheses denote number of subjects with data in each treatment condition*.

c *Included (attempted) homicide, aggravated assault, (aggravated) robbery, (attempted/aggravated) rape, and (aggravated) arson. However, no participants were reconvicted of (attempted) homicide or (attempted/aggravated) rape during follow-up*.

d *Defined as total number of all new counts across all court sentencing occasions during the entire follow-up*.

### Follow-Up: Registered Criminal Recidivism at 12 and 24 Months

Violent reconviction rate differences were small but non-significantly higher for iCBT+TAU youth at 12 months (34 vs. 23%, *d* = 0.30, 95% CI: −0.24 to 0.84) and 24 months (50 vs. 40%, *d* = 0.23, 95% CI: −0.25 to 0.72). Similarly, any reconviction differences at 12 and 24 months were marginal to small and non-significant.

There were small-sized, non-significant differences in number of offense counts favoring TAU-only over iCBT+TAU youth at 12 months (Mann-Whitney *U* = 738.00, *p* = 44, *d* = 0.17), and at 24 months (*U* = 712.00, *p* = 0.32, *d* = 0.22).

We used Cox proportional hazards regression to compare recidivism rates for iCBT+TAU and TAU-only subjects for the entire follow-up period (*M* = 42 months, *SD* = 8, range 27–54 months). Controlling for five small-sized covariate differences at baseline, iCBT+TAU compared to TAU-only participants had slightly, non-significantly higher risk of violent recidivism [adjusted Hazard Ratio (aHR) = 1.57; 95% CI: 0.78 to 3.16] and negligibly lower for any recidivism (aHR = 0.89; 95% CI: 0.51 to 1.54), respectively. Corresponding estimates for the initial, unadjusted Cox regression model were essentially the same (data not shown).

### Completer Analyses

Despite the best efforts of the contributing therapists and their supervisor, only 15 of 38 youths (39%) randomized to iCBT finished enough sessions (15 or more) to be considered completers. Regarding baseline characteristics likely to affect treatment adherence, iCBT completers were moderately but non-significantly older than TAU-only youth (*M* = 18.1; *SD* = 1.1 vs. *M* = 17.7, *SD* = 0.7; *d* = 0.51; 95% CI: −0.09 to 1.10), and had substantially less ADHD symptoms (*M* = 2.00, *SD* = 2.36 vs. *M* = 3.58, *SD* = 1.99, *d* = −0.76; 95% CI: −1.36 to −0.15) and oppositional defiant symptoms than TAU youth (*M* = 2.33, *SD* = 2.47 vs. *M* = 3.91, *SD* = 2.54, *d* = −0.63, 95% CI: −1.22 to −0.03). Finally, iCBT completers had moderately but non-significantly more SAVRY protective factors compared to TAU-only youth (*M* = 3.73, *SD* = 1.22 vs. *M* = 2.84, *SD* = 1.82, *d* = 0.53, 95% CI: −0.07 to 1.12). There were no other meaningful differences between iCBT completers and TAU-only youth on the remaining baseline measures.

Regarding criminal reconvictions, 20% (*n* = 3) of completing iCBT participants vs. 23% (*n* = 10) of TAU-only youth recidivated in a violent crime within 12 months (*d* = −0.11, 95% CI: −0.94 to 0.72). Corresponding figures for any crime within 12 months was 60% for iCBT completers (*n* = 9) and 65% (*n* = 28) for TAU-only participants (*d* = −0.14, 95% CI: −0.88 to 0.61). At 24 months, 33% (*n* = 5) of iCBT completers vs. 40% (*n* = 17) of TAU-only participants had been reconvicted for a violent crime (*d* = −0.16, 95% CI: −0.92 to 0.59). Correspondingly, within 24 months, 53% (*n* = 9) of iCBT completers and 74% (*n* = 32) of TAU-only participants had recidivated in any crime (*d* = −0.40, 95% CI: −1.16 to 0.36).

Finally, for the full follow-up, Cox regression modeling suggested marginal to small, non-significant, risk reductions for violent reconvictions (aHR = 0.85; 95% CI: 0.28 to 2.55) or any reconviction (aHR = 0.64; 95% CI: 0.28 to 1.45) when comparing iCBT completers with TAU-only participants.

## Discussion

Concerned by the limited support for the effectiveness of available group-based psychological interventions in residential care for serious violent young offenders, we investigated the potential effectiveness of the *addition of an individualized CBT intervention* to TAU. A nationwide consecutive sample of 81 youths were randomized to iCBT+TAU or TAU-only in a five-site ecological setting in Sweden. There were three main findings. First, we found substantial pre- to post-treatment improvements on self-reported conduct problems, aggression scores and antisocial cognitions for both iCBT+TAU and TAU-only youth, but no meaningful differences between treatment arms. Second, in intent-to-treat analyses, we were unable to statistically ascertain risk-reducing effects of iCBT treatment on aggression scores at 12 months or on registered reconvictions in violent or any crime at fixed 12- and 24-month follow-ups. Neither did we find any risk-reductions effects of iCBT when looking at the full follow-up period, or on number of conviction counts at 12- and 24-months following release to the community. Third, although complementary per-protocol analyses suggested negligible to small effects favoring iCBT *completers* over TAU youth, these comparisons were also non-significant.

We conclude that an individualized CBT intervention for medium-to-high risk young male violent offenders in residential treatment, focusing on cognitive self-control, and relapse prevention, was insufficient to reduce aggression and criminal reconvictions over and above group-based TAU treatments. iCBT included components known to be associated with positive outcomes, including relapse prevention, focus on interpersonal skills, and homework assignments [e.g., (5, 55)], but may have been insufficiently long and intensive to have desired impact. No feasibility or pilot studies with iCBT were done before the RCT. Because the full iCBT protocol had not been tested beforehand, there is a risk that the manual was not instructive and supportive enough for the therapists or that the training and supervision of therapists was inadequate. Further, serious violent young offenders are characterized by many criminogenic risk factors across multiple risk areas; hence, this population probably needs several different but integrated interventions to prevent reoffending and establish a prosocial life [cf. (12)]. For instance, pharmacological treatment might help clients with impulsivity and emotional dysregulation, and vocational training and social support may be necessary to establish and maintain a prosocial lifestyle. Finally, the study design with both treated and control youth in the same residential homes and cross-facility variability in TAU treatment may have hindered satisfactory integration of iCBT and TAU interventions. This, in turn, may have resulted in poorer iCBT effectiveness.

Potentially effective pharmacological treatment with central stimulants for ADHD, mood stabilizers, and medications against drug craving are substantially more common in residential care today than in 2003–2006. Unless considered in study design and analyses, their use may complicate inferences about possible effects of psychological interventions, for example by leveling out the outcomes of experimental and control groups. Although other studies reveal small possible changes in the prevalence of substantial psychiatric (co)morbidities over time [e.g., (56)], we are unaware of temporal sample composition changes that could affect external validity of the present results.

Proper program implementation is crucial for effectiveness [e.g., (57)]. For example, Helmond et al. (58) argued that the low to moderate treatment integrity they found for the EQUIP program, a CBT intervention common in juvenile correctional facilities in North America, Australia, and Europe (59) could, at least partly, explain the lack of recidivism-reducing effect in their Dutch study. Systematic use of feasibility or pilot studies could be a way to ensure that clinical settings are ready for an effectiveness trial (60). Other methods for monitoring fidelity during implementation and evaluation of interventions in real-world settings include monitoring clients' homework production, and video- or audio monitoring of treatment sessions to assure therapist adherence to manuals, protocols, and treatment principles. In an RCT of Multisystemic Therapy (MST) effectiveness (61), for non-residential adolescent offenders in Sweden, Sundell et al. (62) found no significant post-treatment differences in problem behaviors between MST and TAU participants. They also reported lower scores than previous studies on the MST treatment fidelity measurement (TAM). Since effectiveness differences across participating sites were unrelated to TAM scores, these authors did not attribute the lack of MST effectiveness solely to site effects and program immaturity in terms of TAM-scores. Instead, Sundell et al. (62) suggested the potential validity threat of *TAU variability* (63–66) as an alternative explanation for similar improvements among treatment and control subjects. On a related note, transfer study investigators of MST, an intervention for youth developed in the USA (62, 67) also argued that TAU may be more potent in countries with stronger focus on tax-funded, general child welfare systems such as Sweden and some other European countries. A stronger relative effect of TAU in Sweden would make it more difficult to uncover potential effects of a complementary intervention like iCBT. Since TAU in the current study involved incarceration for a substantial time, the iCBT intervention may have been too short and not intensive enough to exert impact beyond TAU.

Finally, the observed pre- to post-intervention improvements in this study were not reflected in the high recidivism rates found for both groups. This aligns with previous findings that short-term reductions in individual criminogenic risk factors often fail to produce reduced recidivism after release (68–70). It may be that maintenance of possible individual changes achieved in treatment requires sufficient support following release also on reasonable conditions in terms of housing, studies or work, and prosocial interpersonal relations.

## Limitations and Strengths

Even with the rather brief iCBT intervention, less than half of those randomized eventually received the full intended dose. Consistent with prior studies [e.g., (71–73)], participants dropping out of iCBT treatment before completion of the full treatment (15+ sessions) had substantially higher risk of reoffending compared to TAU-only controls (data not shown). This finding suggests *dose and dropout* issues, or the importance of receiving an adequate, planned amount of treatment to avoid the risk of harming vulnerable individuals. We had no systematic measurements of initial motivation to engage in treatment; a predictor of treatment attrition among young offenders (74). Notably, treatment completers had significantly less (medium effects) ADHD and oppositional defiant symptoms compared to non-completers. This suggests that such symptoms may increase attrition risk and need consideration in treatment planning. The iCBT manual provided specific approaches for handling dropout risk, for instance by using motivational interviewing strategies with youth signaling lowered motivation. However, despite being addressed in continuous supervision of iCBT treatment providers, this may have been insufficient to secure treatment completion.

Second, systematic reviews of treatment of young offenders suggest that effectiveness studies (i.e., in regular clinical settings) more often suffer from attrition, insufficient descriptions of implementation or poor quality of the latter than do efficacy studies[in more specialized, research-oriented settings; e.g., (17, 75)]. Suboptimal *treatment integrity* could be a limitation also in this study. Contributing iCBT therapists were expected to participate in repeated supervision in person and by email and were encouraged to contact the supervisor whenever needed. However, perhaps inevitably with high-risk serious violent youth clients, some of them experienced difficulties with adhering to the treatment process. Based on the suggested non-significant trend toward marginal or small positive effects for iCBT completers compared to TAU-only controls, treatment non-completion may have influenced overall results.

Third, designing studies based on well-informed, careful calculation of *statistical power* is important to avoid random errors due to data variability [e.g., (76)]. Although we tried to balance the risk of type I and II errors by using the less conservative alpha level of 0.10, our study was underpowered, primarily due to an initial overestimation of possible treatment effects and client attrition. Nevertheless, the iCBT effects suggested here, negligible or favoring the TAU condition, argue against further testing of the current iCBT format as an add-on to group-based interventions.

Strengths of the study include high ecological validity (conducted in a real-world clinical setting, with few exclusion criteria and with local therapists), contrasting iCBT with an active and relevant comparator (TAU) used in most youth residential treatment facilities in Sweden, and including multiple sources of information; masked clinician ratings, self-reports, and nationwide crime register data.

## Conclusion

A complementary, individualized 15–20-session CBT intervention focusing problem-solving, cognitive self-control, and relapse prevention for serious, young male violent offenders in residential treatment in Sweden was insufficient to reduce aggression and criminal reconvictions during the 24 months following release, beyond group-based TAU. Intent-to-treat effect estimates were imprecise due to restricted sample size and considerable attrition. However, suggested negligible or negative effects argues against further testing of the iCBT format as a complement to evidence-based group interventions, although firm conclusions cannot be drawn given the present limitations.

Besides drawing attention to the relative effectiveness of comparison interventions, our findings underscore the need for more effective, comprehensive, and individualized interventions for serious young violent offenders in residential treatment. Further integration of psychological and psychiatric treatment [cf. (56)], well-performed program implementation, and effective strategies for maintaining treatment integrity are likely to benefit these vulnerable and costly high-risk youth and society alike.

## Data Availability Statement

The dataset presented in this article is not readily available since it contains sensitive details about the participants. Requests to access the dataset should be directed to Martin Lardén, martin.larden@kriminalvarden.se.

## Ethics Statement

The study was reviewed and approved by the Regional Ethics Review Board in Stockholm, Sweden. Written informed consent to participate was provided by the youth himself and, for participants younger than 18 years, also from parent(s)/legal guardians.

## Author Contributions

ML and NL designed the study. ML coordinated the data collection, conducted the data analyses, and drafted the initial manuscript. All authors reviewed the manuscript for important intellectual content and helped shape interpretations and the final manuscript.

## Conflict of Interest

The authors declare that the research was conducted in the absence of any commercial or financial relationships that could be construed as a potential conflict of interest. ML designed the CBT intervention, but has not made economic or other gains from royalties, training, or consulting regarding the model.

## Publisher's Note

All claims expressed in this article are solely those of the authors and do not necessarily represent those of their affiliated organizations, or those of the publisher, the editors and the reviewers. Any product that may be evaluated in this article, or claim that may be made by its manufacturer, is not guaranteed or endorsed by the publisher.

## Footnotes

^1^To better capture sexually abusive behavior without explicit force, we added “has had sex with someone unwilling by using pressure or drugs” as an additional item to complement the original aggressive CD criterion “has forced someone into sexual activity”.
