# Supplementary material for: Effectiveness of an Individual Cognitive-Behavioral Intervention for Serious, Young Male Violent Offenders: Randomized Controlled Study With Twenty-Four-Month Follow-Up
Source: Front Psychiatry. 2021 Aug 2;12:670957. doi: 10.3389/fpsyt.2021.670957 (PMC8365084; doi:10.3389/fpsyt.2021.670957)
Supplement: Supplementary file 1 [file Data_Sheet_1.docx]

**Supplement 1:**

**Brief description of the iCBT intervention for antisocial youth originally published in Swedish (Lardén 2002; 2003).**

**Case formulation**

Before the treatment starts (according to phases I-IV below), the iCBT therapist makes a case formulation serving as a general treatment plan. The case formulation is based on a structured assessment of criminogenic risk factors, needs and protective factors that should be addressed to support a prosocial development.

The case formulation plan includes the following areas:

1. **Violent offending:** Description of the violent offence(s) included in the current conviction.
2. **Personal history:** Includes general childhood conditions such as immigrant status, family constellation, parental substance abuse, and antisocial development.
3. **Individual need factors:** Encompasses five risk- and needs factor areas: criminal history, antisocial attitudes and values, alcohol and illicit drug use, temperament and personality and psychiatric health (cf. Andrews & Bonta, 1998).
4. **Interpersonal relationships:** Including family members, peers, teachers etc.
5. **School and education:** Academic experiences and achievement.
6. **Assets and resources:** Includes individual protective factors, social support and other factors that may help establish and maintain a prosocial life.
7. **Other important information** that could affect treatment is noted, for example conduct problems in the residential setting, threats from other youth etc.

**Treatment phases**

After the establishment of a case formulation and general treatment plan, iCBT treatment follows the phases outlined below:

*Phase I: Motivation and goal planning*

Proactive motivational work is a key ingredient in iCBT, and the therapist continues throughout treatment to motivate the youth to participate actively in the change process. Time is also spent on helping the youth to formulate his own treatment goals.

*Phase II: Problem-solving*

This phase consists of the problem-solving process and interpersonal skills training of a “Middle Way” model (Lardén, 2002) inspired by Buddha’s middle way. Introduced techniques constitute basic strategies throughout treatment and support acting in ways that are neither passive nor aggressive but instead assertive and promoting of positive interpersonal interactions.

*Phase III: Cognitive self-control*

This phase aims to elucidate the connection between thinking patterns and behaviors, specifically how antisocial cognitive distortions and other rigid thought patterns make individuals “fool themselves” to commit destructive or antisocial acts. Important theoretical starting points are Dodge and colleagues’ theories of social information processing (1990) and Novaco’s model of anger (1975).

*Phase IV: Relapse prevention*

This phase is about learning how to recognize risk situations, to analyze them with the help of problem-solving and to practice coping skills to manage these risk situations. Formulation of specific action plans are made jointly by the youth and therapist. The phase is modeled on Marlatt and Gordon’s relapse prevention strategies (1985).

**References**

Andrews, D. A., & Bonta, J. (1998). *Psychology of criminal conduct (2nd ed.)*. Cincinnati, OH: Anderson Publishing.

Dodge, K. A., Price, J. M., Bachorowski, J.-A., & Newman, J. P. (1990). Hostile attributional biases in severely aggressive adolescents. *J Abnorm Psychol, 99,* 385–392.

Lardén, M. (2002). *Från brott till genombrott. Kognitiv beteendeterapi för tonåringar med psykosociala problem.* (in Swedish: From law-breaking to break-through. Cognitive behavior therapy for adolescents with psychosocial problems). Stockholm: Gothia.

Lardén, M. (2003). *Terapeutmanual för IKBT.* (in Swedish: *Therapist manual for ICBT*). Unpublished manual. Stockholm: Karolinska institutet.

Marlatt, G. A. & Gordon, J. R. (1985). *Relapse prevention: Maintenance strategies in the treatment of addictive behaviors.* New York: Guilford.

Novaco, R. (1975). *Anger control: The development and evaluation of an experimental treatment.* Lexington, DC, USA: Heath.
